# Endoscopic surgery combined with titanium mesh for infantile solitary orbital myofibroma: a case report

**DOI:** 10.3389/fped.2025.1602242

**Published:** 2025-06-18

**Authors:** Ligang Jiang, Xin Jiang, Wencan Wu, Fangzheng Jiang

**Affiliations:** ^1^ Department of Ophthalmology, Quzhou Affiliated Hospital of Wenzhou Medical University, Quzhou People’s Hospital, Quzhou, Zhejiang, China; ^2^ Department of Economics and Management, Quzhou College of Technology, Quzhou, Zhejiang, China; ^3^ The Eye Hospital, School of Ophthalmology & Optometry, Wenzhou Medical University, Wenzhou, China; ^4^ Oujiang Laboratory (Zhejiang Lab for Regenerative Medicine Vision and Brain Health), Wenzhou, Zhejiang, China; ^5^ Wenzhou Institute, University of Chinese Academy of Sciences, Wenzhou, China

**Keywords:** infantile tumors, solitary orbital myofibroma, spindle cell tumor, immunohistochemistry, endoscopic surgery, titanium mesh implantation, case report

## Abstract

**Background:**

To report the clinical features, misdiagnosis process and minimally invasive treatment experience of endoscopy combined with titanium mesh in a 3-year-old infant with isolated orbital myofibroma, and to discuss the key points of differential diagnosis and treatment strategy.

**Case report:**

A 3-year-old male patient presented with progressive swelling of the left lower eyelid for 1 month, without eye redness, eye pain, diplopia, or ocular motility disturbance. There was no significant family history or past medical history. Ophthalmic examination revealed visual acuity of 0.5 in the right eye and 0.6 in the left eye. Swelling was observed in the right face and the lower eyelid, with a palpable mass that was well-mobile, firm in texture, and non-tender. The ocular positions were normal with regular motility, while anterior segment examination and fundoscopy showed no abnormalities. Imaging information indicated a well-defined oval mass (1.5 cm × 2.1 cm) in the infraorbital foramen area of the anterior wall of the right maxillary sinus, accompanied by erosion and destruction of the anterior wall of the maxillary sinus. Because of the imaging features and frozen section were highly similar to those of schwannoma, both presenting as well-defined spindle cell tumors, a misdiagnosis of “right orbital schwannoma” was identified. However, that misdiagnosis did not alter the surgical approach. We performed endoscopic microsurgery to achieve precise resection and avoid damaging normal tissues. Meanwhile, a titanium mesh was implanted to reconstruct the orbital bone defect, restoring its anatomical structure and function. Intraoperative frozen section showed a spindle cell tumor, which tended to be diagnosed as schwannoma. Postoperative immunohistochemistry indicated SMA (+), Calponin (+), Ki-67 (+, proliferation index of 20%), Desmin (−), S-100 (−), CD34 (−), CK (−), leading to the final diagnosis of infantile solitary orbital myofibroma.

**Conclusion:**

Infantile orbital myofibroma is clinically rare and frequently misdiagnosed as schwannoma, which requires immunohistochemical and molecular genetic testing for definitive diagnosis. Endoscopic minimally invasive techniques demonstrate significant advantages in preserving normal tissues. Titanium mesh can effectively reconstruct orbital bone defects and restore anatomical structure and function. However, long-term follow-up is required to monitor its potential impact on maxillofacial development in infants and young children.

## Introduction

Infantile myofibroma is a rare benign myofibroblastic tumor predominantly affecting male infants under 2 years of age (1). It typically manifests in the head and neck region, including the scalp, forehead, parotid area, and oral cavity. The tumor is associated with diverse clinical presentations and potential severe complications, including multiorgan involvement and even mortality (2).

Infantile myofibroma can be categorized into solitary, multicentric, and extensive types. Solitary lesions are the most common, accounting for approximately half of all cases (1). Most cases exhibit spontaneous regression, and orbital invasion is rarely observed (3–5). Shields et al. (6) did not document any cases of orbital myofibroma in their series of 645 orbital tumors. Similarly, Kodsi et al. (7) reported only one case of orbital myofibroma in their comprehensive review of 340 orbital tumor cases. Orbital myofibroma typically comprises spindle-shaped fibrocytes and myofibroblasts, exhibiting an invasive growth pattern. Due to its diverse clinical manifestations, it is prone to misdiagnosis.

This case report discusses a 3-year-old infant with solitary orbital myofibroma. Initial clinical signs, imaging examination and frozen section suggested orbital schwannoma, which led to misdiagnosis as “right orbital schwannoma”, but did not affect the surgical method. We used endoscopic microsurgery to achieve accurate resection, avoiding the damage of normal orbital tissue. Titanium mesh was implanted to repair orbital bone defect and restore its anatomical structure and function. After surgery, isolated orbital myofibroma in infants was confirmed by immunohistochemical analysis.

## Case presentation

The patient was a 3 year old male who presented with progressive swelling of the lower eyelid of the left eye for 1 month. The patient had no redness, pain, diplopia, or eye movement disorder, no significant family history or previous medical history, and no intervention or treatment in other hospitals.

Ocular examination revealed visual acuity of 0.5 in the right eye and 0.6 in the left eye. There was noticeable swelling in the right facial area and lower eyelid, with a palpable, firm mass that was mobile and non-tender. The position of both eyes was normal, and ocular motility was intact. Anterior segment examination and fundoscopy showed no abnormalities.

**Figure 1:**
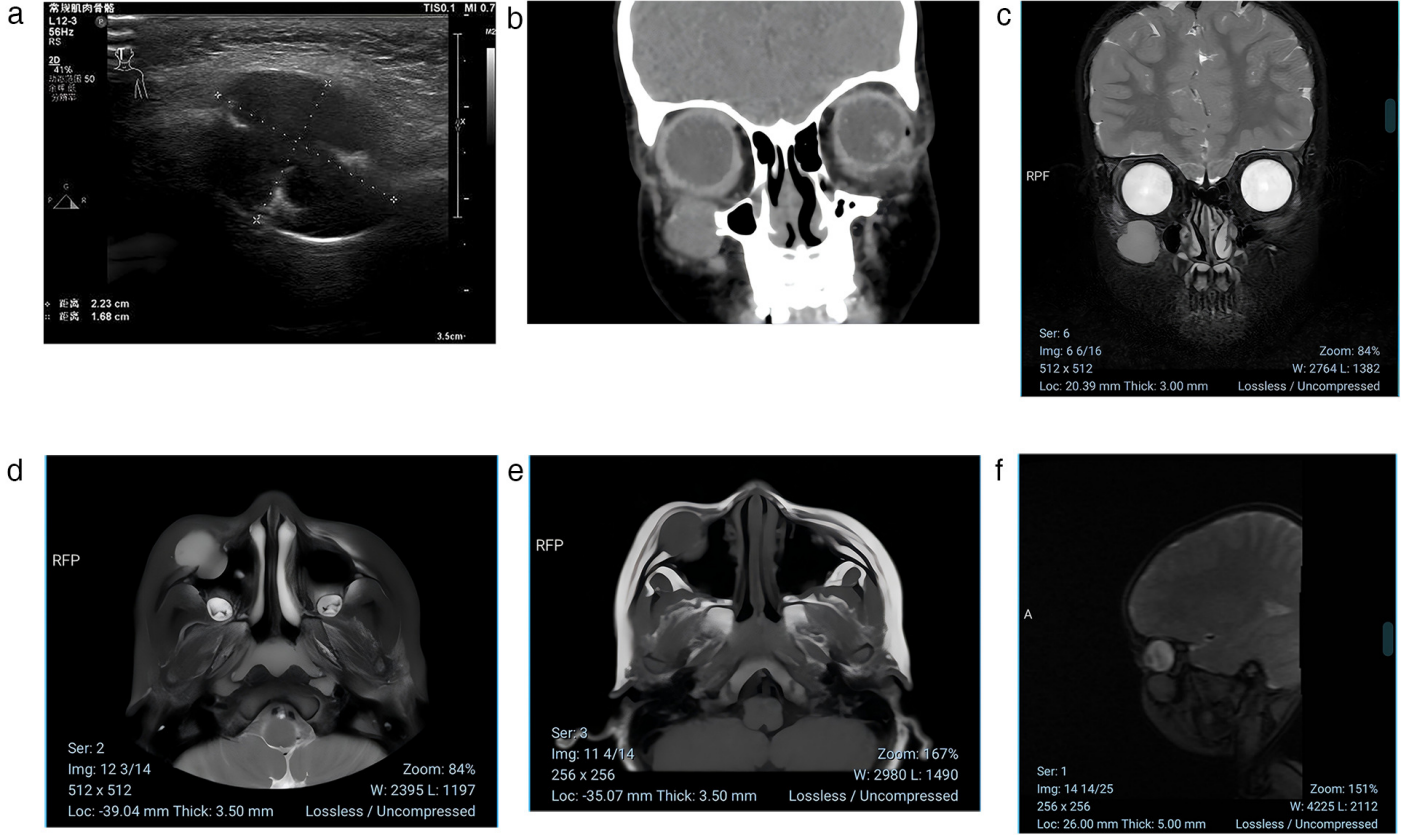
**(a)** Ultrasound examination indicated a mass in the anterior wall of the right maxillary sinus. The lesion measures approximately 2.23 cm × 1.68 cm in maximum diameter, demonstrating clear boundaries and heterogeneous echogenicity. **(b)** A coronal CT scan of the paranasal sinuses revealed a mass in the area of the right maxillary sinus anterior wall. Coronal CT scan showing an oval-shaped isodense lesion in the region of the right maxillary sinus anterior wall and infraorbital canal. The lesion measures approximately 1.5 cm × 2.1 cm, with well-defined margins and uniform density (CT value approximately 30–35 HU). Notable expansion is observed, with protrusion into both the maxillary sinus cavity and subcutaneous tissue, accompanied by localized bone destruction of the anterior wall of the maxillary sinus. **(c–f)** MRI scan showing an oval-shaped abnormal signal in the region of the right maxillary sinus anterior wall and infraorbital canal. On T2-weighted imaging (T2WI) and FLAIR sequences, the lesion appears hyperintense, while it is hypointense on T1-weighted imaging (T1WI). The lesion measures approximately 1.5 cm × 2.1 cm and exhibits significant heterogeneous enhancement on post-contrast scans.

Ultrasound examination (Figure 1a) indicated a mass in the anterior wall of the right maxillary sinus, raising the suspicion of a neurogenic tumor. A coronal CT scan of the paranasal sinuses (Figure 1b) revealed a mass in the area of the right maxillary sinus anterior wall, with bony destruction of the anterior maxillary wall and localized expansile changes. The differential diagnoses included infraorbital schwannoma or hemangioma. An enhanced orbital MRI (Figures 1c–f) confirmed the presence of a mass in the same region, suggesting a neurogenic tumor, most likely a schwannoma. Based on the clinical manifestations and imaging findings without history of ocular trauma or surgery, the patient was initially diagnosed with right orbital schwannoma. Preoperative evaluation ruled out any surgical contraindications.

**Figure 2:**
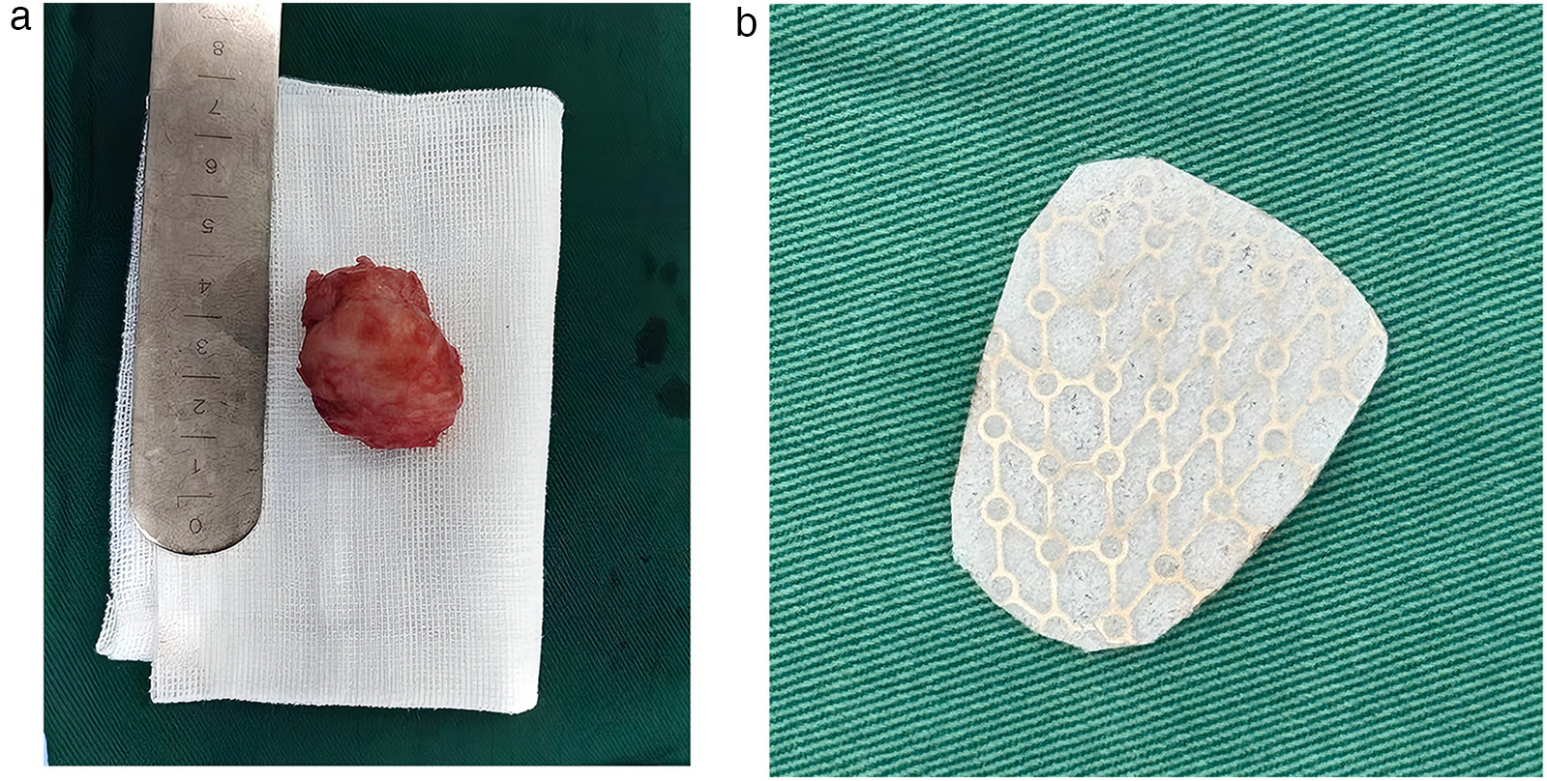
**(a)** Intraoperative image. The tumor was approximately 3.1 cm × 2.4 cm × 2.3 cm in size and had a hard fibrous texture. **(b)** Intraoperative implantation of a titanium mesh to reconstruct orbital defects and restore anatomical structures.

**Figure 3:**
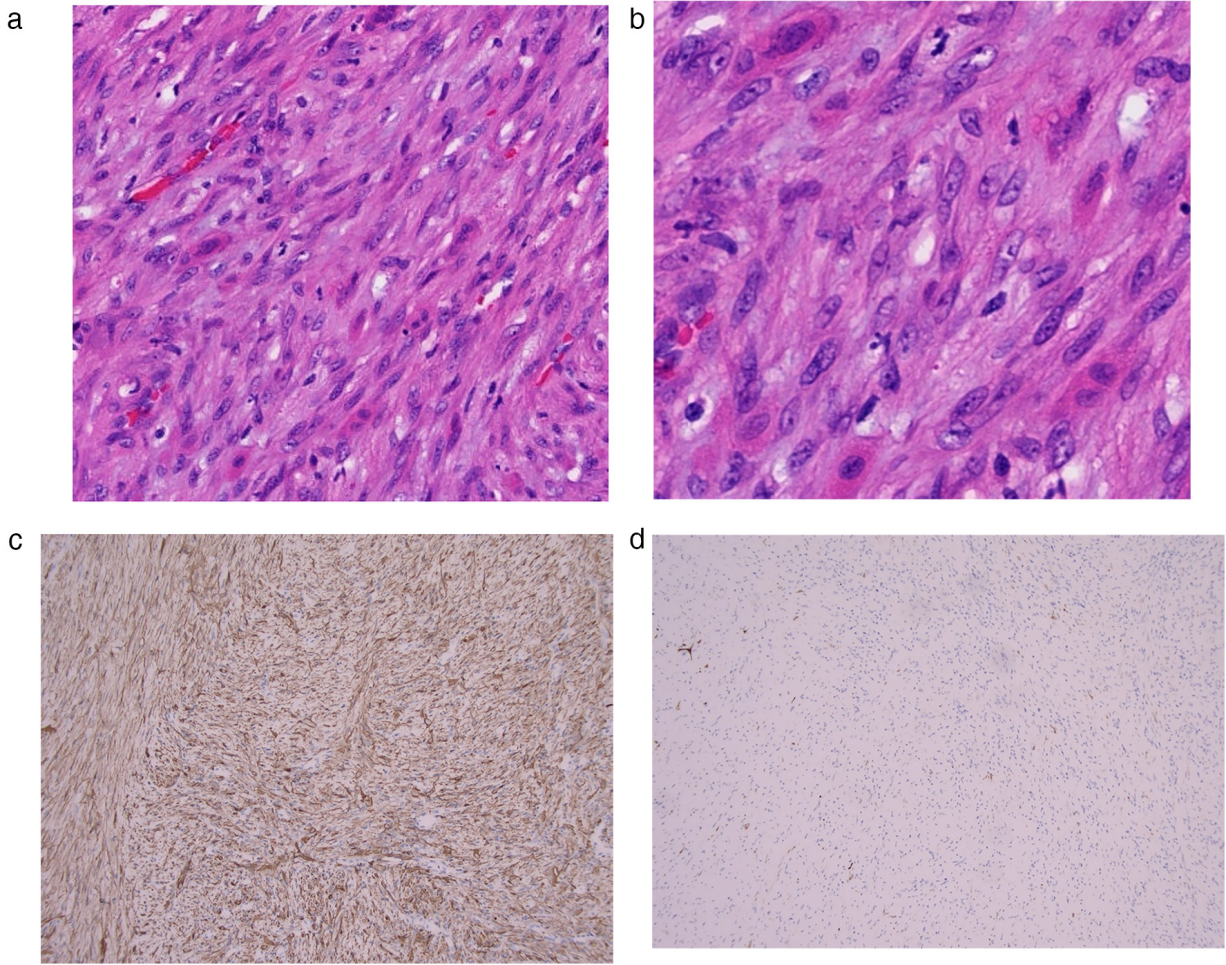
**(a)** 10× spindle cell tumor exhibiting fascicular and whorled growth patterns. Abundant intercellular collagen fibers form dense fibrous stroma, which is consistent with a lesion of mesenchymal origin and were misdiagnosed as a neurogenic tumor. **(b)** 20× It has uniform chromatin distribution, plump oval nuclei, scant cytoplasm with mildly eosinophilic. No areas of necrosis within the tumor, and no mitotic activity is observed. **(c,d)** Immunohistochemical results. **(c)** SMA (+). **(d)** Calponin (+). The combined morphological and immunohistochemical features confirms the diagnosis of solitary orbital myofibroma.

After successful general anesthesia, the child was placed in a supine position, and the area was routinely disinfected and draped. Under the assistance of an endoscope, an orbital mass resection was performed through the skin approach of the right lower eyelid. The orbicularis oculi muscle was dissected layer by layer to fully expose the periosteum on the lower margin of the orbit. A dissection plane was established between the periosteum and the orbital tissue. Under the clear vision of the endoscope, the mass was carefully dissected along its boundary. During the operation, gentle and meticulous movements were made to completely remove the mass. Subsequently, the range and depth of the bone defect were precisely measured, and the titanium mesh was accurately trimmed based on the measurement results to perfectly fit the anatomical curvature of the orbital wall (Figure 2). The trimmed titanium mesh was implanted into the bone defect to maintain the normal shape and structure of the orbit (Figure 2). Throughout the operation, close attention was paid to and precise protection was provided for the surrounding arterial branches and neural tissues to avoid damage. Intraoperative rapid frozen section analysis (Figure 3) showed spindle cell tumor. Based on the intraoperative findings and frozen section results, it was initially considered to be a schwannoma. The final diagnosis will be confirmed by routine histological examination and immunohistochemical analysis. Postoperative immunohistochemical analysis (Figure 3) showed SMA (+), Calponin (+), Ki-67 (+, proliferation index of 20%), Desmin (−), S-100 (−), CD34 (−), and CK (−), which confirmed the diagnosis of typical infantile solitary orbital myofibroma rather than neurilemmoma. The child recovered well after surgery, with no exophthalmos and normal eye movement in all directions. At the 3-month follow-up, there was no eye discomfort, the visual acuity was the same as before the operation, the titanium mesh was not displaced, and there was no evidence of systemic or visceral involvement.

**Figure 4:**
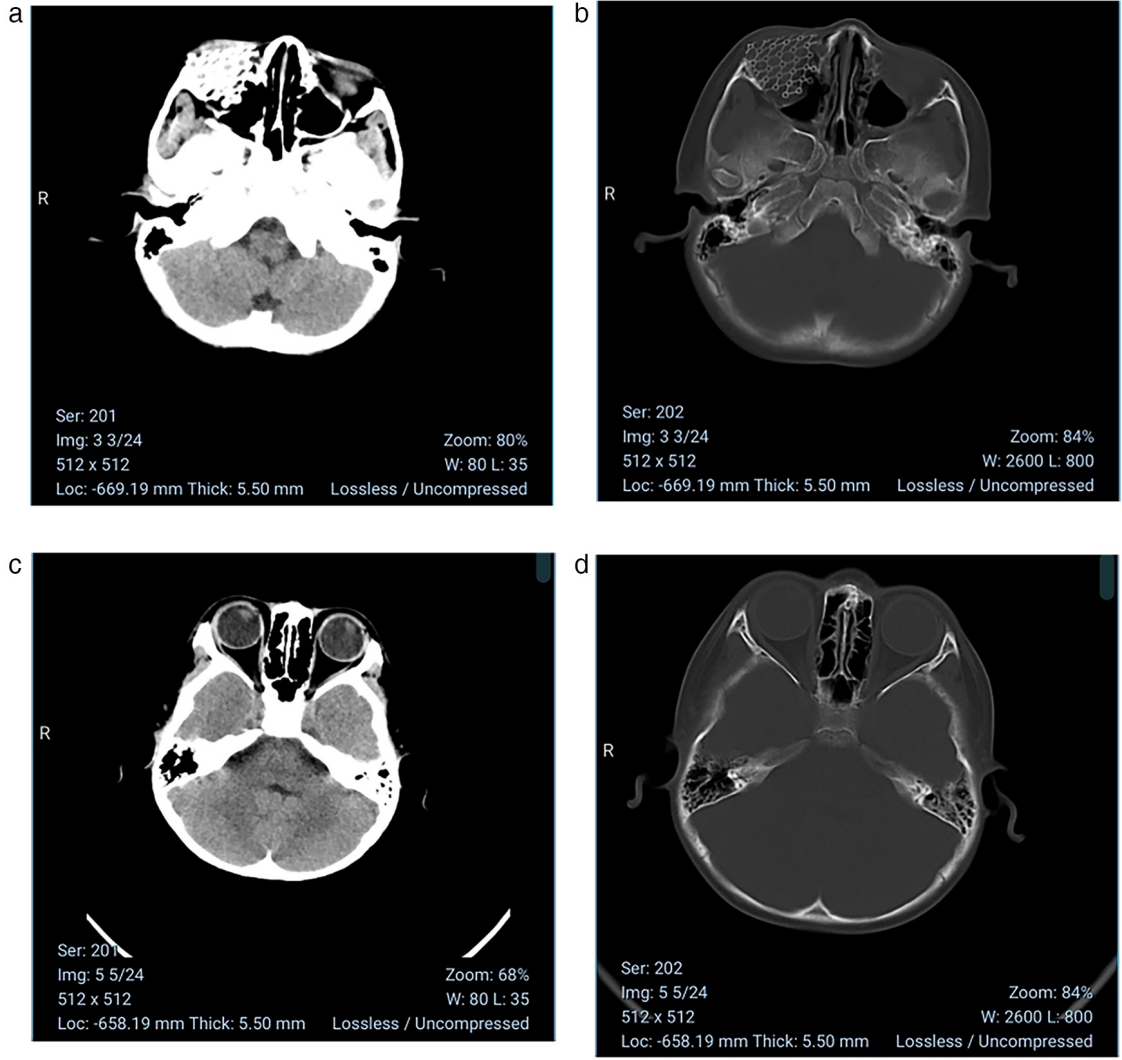
**(a,b)** At the 3-month postoperative follow-up, imaging demonstrated stable positioning of the titanium mesh within the orbit without any displacement or infection. **(c,d)** The patient has uneventful recovery with normal ocular motility in all directions after the surgery, no proptosis or displacement of the eyeball and same preoperative visual acuity.

## Discussion

Infantile solitary orbital myofibroma is a rare benign soft tissue tumor that typically occurs in the head and neck region of infants. Due to its rarity, orbital myofibroma is prone to clinical misdiagnosis, potentially leading to over treatment or delayed intervention. Differentiating infantile solitary orbital myofibroma from orbital schwannoma can be particularly challenging, as both tumors share several clinical, radiological, and histopathological similarities, making misdiagnosis possible without thorough examination.

In this case report, a 3-year-old child presented with right lower eyelid swelling for one month. Initial imaging studies strongly suggested schwannoma, and intraoperative frozen section pathology also supported a spindle cell tumor, most likely a schwannoma. The definitive diagnosis of myofibroma was ultimately confirmed through the results of immunohistochemical analysis showing SMA (+), Calponin (+), Ki-67 (+, proliferation index of 20%), Desmin (−), S-100 (−), CD34 (−), CK (−).

The misdiagnosis in this case initially arose due to the overlapping imaging features of myofibroma and schwannoma on ultrasound, CT, and MRI. Both kind of the tumors typically present as well-defined orbital masses (8), appearing as oval, round, or irregular lesions with heterogeneous internal echoes or signals, which may reflect possible cystic degeneration or necrosis within the tumor. These shared characteristics can lead to ocular motility restriction, eyelid swelling, painless proptosis, or visual impairment. The clinical, radiological, and frozen section features of these two tumors are remarkably similar, and both are common orbital spindle cell tumors (9–11). While schwannomas can occur at any age but are relatively more common in adults, pediatric cases have been documented. Nagashima et al. (12) reported a confirmed case in a 5-year-old male through histopathological and immunohistochemical analysis. Kamphausen et al. (13) described a case of a 6-year-old boy who presented with acute unilateral exophthalmos, decreased vision, tearing, and strabismus. MRI revealed a solid mass within the left orbit, and the postoperative pathology diagnosed it as orbital schwannoma. However, subtle differences exist. Schwannomas typically follow nerve pathways, often demonstrating “dumbbell” or “tail” signs on imaging, while myofibromas exhibit different orbital distribution patterns. On MRI, schwannomas generally show more homogeneous signals, whereas myofibromas may display signal heterogeneity due to their mixed fibrous and myofibroblastic components (14). Despite imaging clues, definitive diagnosis relies on histopathology and immunohistochemistry. In our case, the key to differentiation was the immunohistochemical markers. The immunohistochemical profile for SMA(+), Calponin(+), Ki-67(+) (proliferation index 20%), Desmin(−), S-100(−), CD34(−), CK(−) support the diagnosis of classic myofibroma, while schwannomas typically show S-100 protein positivity, which was negative in this case. A limitation of this case was the lack of FISH testing due to parental refusal, causing the lack of molecular genetic information of the patient.

Surgical excision remains the primary therapeutic approach for infantile myofibroma. The surgical approach is determined by tumor size, location, and extent. Conservative debulking may be considered when complete resection is unfeasible. Shields et al. (15) reported a case of a 3-month-old infant with a painless, osteolytic, superior-medial orbital myofibroma that was resected via a superior-medial orbitotomy. Yazici et al. (16) states a case of an infant with extensive cranio-orbital myofibroma involving both bilateral orbits and ethmoid sinuses, which employed lateral orbitotomy for debulking and biopsy. The case ultimately resulted in mortality at 45 days postoperatively due to complications. The surgical strategy for infantile myofibroma must delicately balance radical resection with functional preservation. Since the tumor may be closely adherent to surrounding tissues, it is crucial to protect important intraorbital blood vessels and neural structures during surgery to achieve complete tumor resection, especially when the lesion involves anatomically complex areas such as the maxillofacial region.

This case innovatively employed the application of endoscopic microsurgery-assisted reconstruction with titanium mesh implantation, characterized by synergistic integration of three-dimensional visualization and millimeter-scale instrument control. Intraoperative high-definition endoscopic assistance enabled successful avoidance of critical vascular and neural structures.In numerous case reports of solitary infantile orbital myofibroma, some researchers have employed endoscopy—assisted surgery. Galassi et al. (17) successfully resected a solitary myofibroma in a 17—month—old girl using an endoscopic approach. Similarly, Amine et al. (3) performed endoscopic resection of a solitary orbital myofibroma in a 6—year—old child. However, the use of endoscopy combined with titanium mesh is relatively rare. In affected infants, the orbital walls are thin, so titanium mesh fixation requires millimeter—level precision to prevent damage to orbital structures.Under endoscopic guidance, we implanted the titanium mesh to ensure accurate coverage of the defect area, repair the orbital bone defect, restore its anatomical structure and function, prevent tissue adhesion and infection, and also improve ocular as well as facial appearance. Although the titanium mesh reconstructed the orbital anatomy, regular imaging follow-up is essential to monitor recurrence and the position of the titanium mesh, with concentrating on the craniofacial development of growing children. Harrison et al. (18) retrospectively analyzed 13 cases of orbital fractures, with an average age of 13 years. Among them, one case used a preformed titanium plate due to a large bone defect. Although the surgery was delayed until 51 days, diplopia recovered rapidly after the operation, and there were no reports of related effects on growth and development.The patient in this case maintained normal ocular movements in all directions, stable preoperative visual acuity, and complication-free recovery with no mesh displacement after the surgery. Compared with traditional open surgery, the utilization of endoscopy offers minimally invasive access and provides a visual surgical field, resulting in milder postoperative discomfort and lower incidence of complications associated with traditional open surgery.

## Conclusion

Emphasizing histopathological details and immunohistochemical markers of tumors are crucial for accurate diagnosis and appropriate treatment. Endoscopic microsurgical techniques enable precise treatment to minimize the damage to normal orbital tissues, significantly reducing the complication rates associated with conventional open surgery. Titanium mesh can effectively reconstruct orbital bone defects to restore anatomical structure and function. However, long-term follow-up is essential to monitor its potential impact on maxillofacial development in infants and young children.

## Data Availability

The original contributions presented in the study are included in the article/Supplementary Material, further inquiries can be directed to the corresponding authors.
